# Supplementary material for: Comparative Analysis of Transcriptomics and Metabolomics Reveals Defense Mechanisms in Melon Cultivars against Pseudoperonospora cubensis Infection
Source: Int J Mol Sci. 2023 Dec 16;24(24):17552. doi: 10.3390/ijms242417552 (PMC10743968; doi:10.3390/ijms242417552)
Supplement: Supplementary file 1 [file ijms-24-17552-s001.zip › Supplemental Figure.pdf]

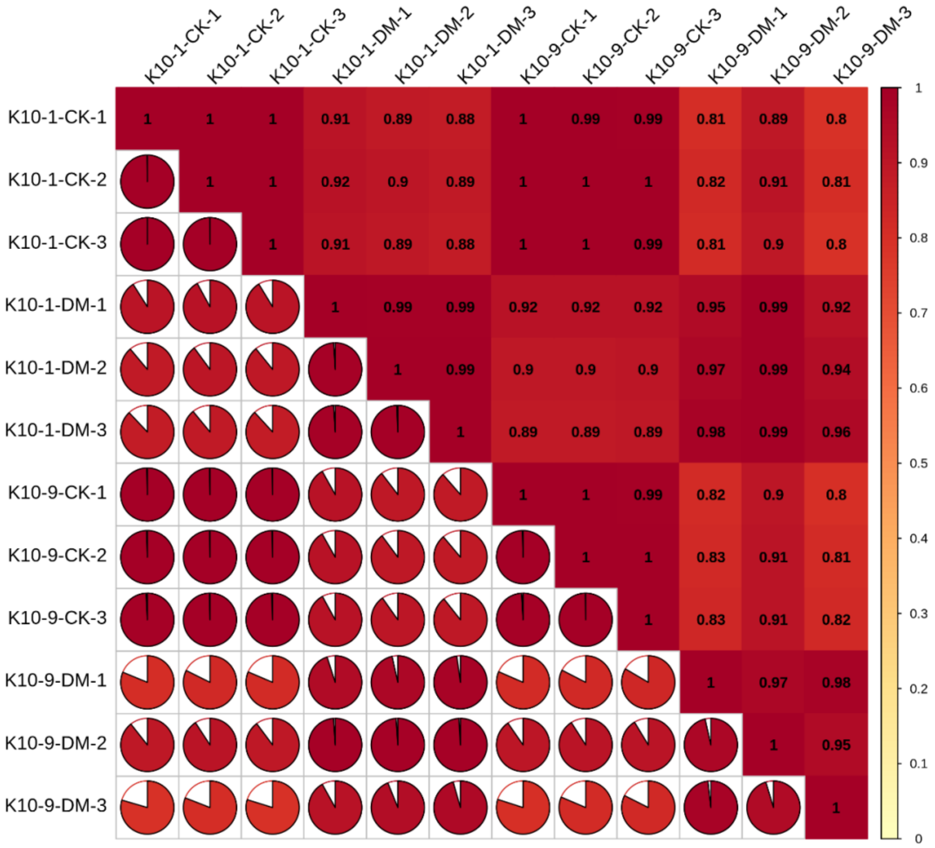

Figure S1 Pearson correlation co-efficiency of RNA-seq data across samples in K10-1 and K10-9.

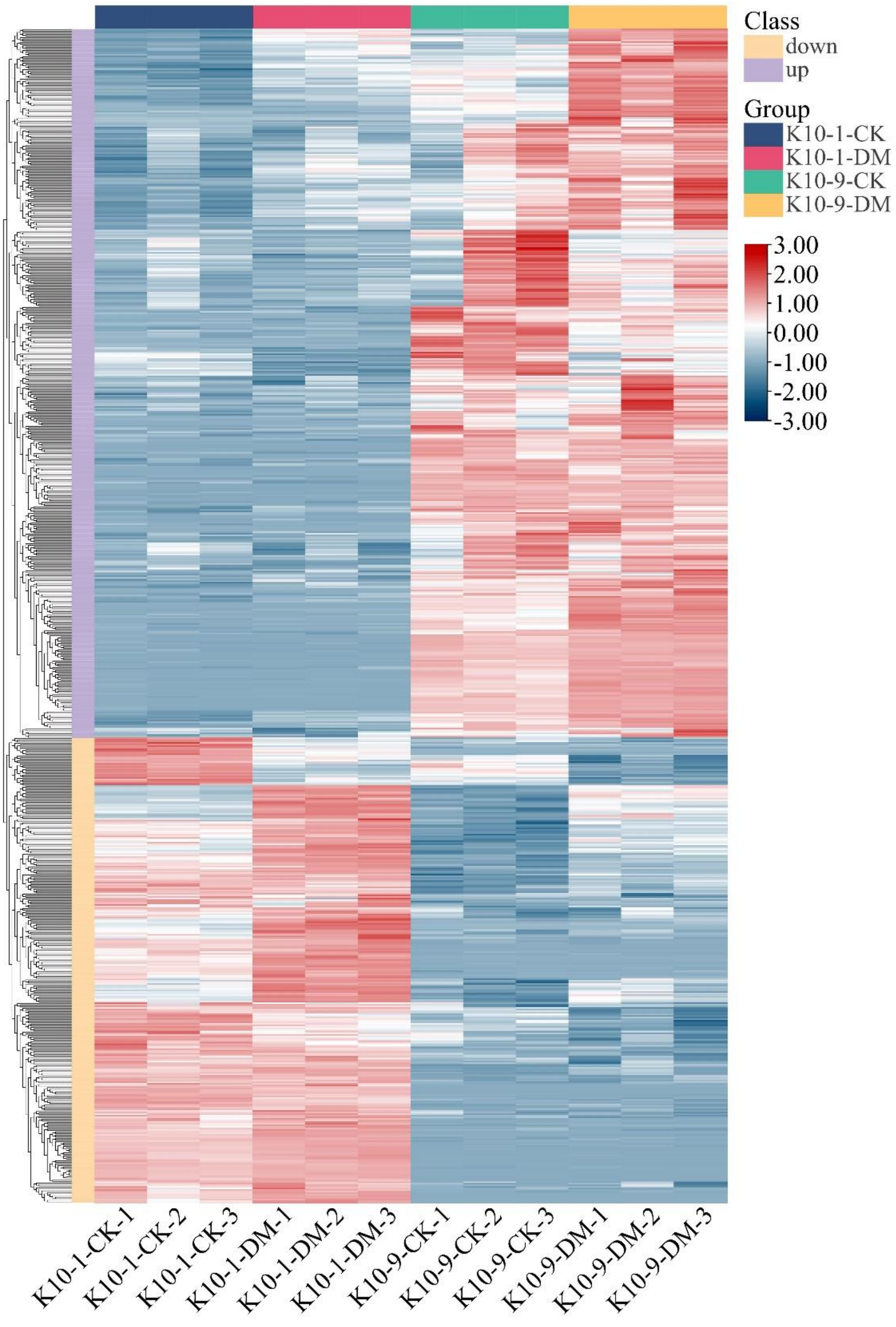

Figure S2 Heat map illustrating the expression patterns of 293 and 447 DEGs in ‘K10-1’ and ‘K10-9’ melon leaves. The expression levels in ‘K10-1’ consistently surpassed and were inferior to those in ‘K10-9’ in the control and treatment conditions, respectively.

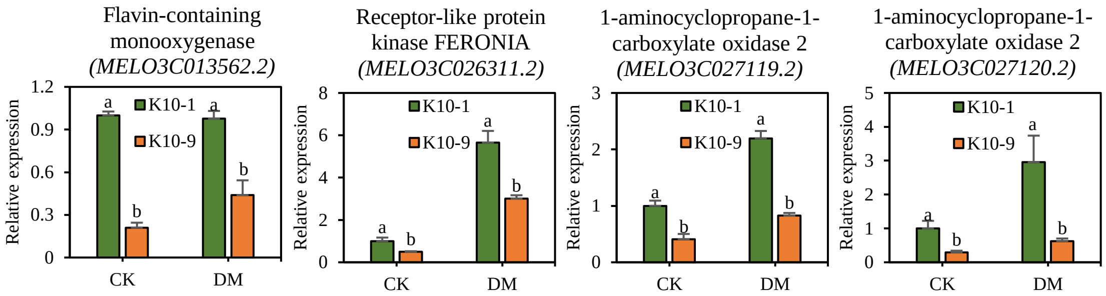

Figure S3 Relative expression levels of four genes related to fungal defense responses in ‘K10-1’ and ‘K10-9’ at 0 hours (CK) and 48 hours post-inoculation (hpi) with *P. cubensis*. The Ct value was normalized to the expression level of the reference gene, *CmADP*. Each error bar denotes the mean ± standard deviation, based on three technical and three biological replicates. Statistically significant differences between groups ($P < 0.05$) are indicated by distinct letters and were determined using the t-test.

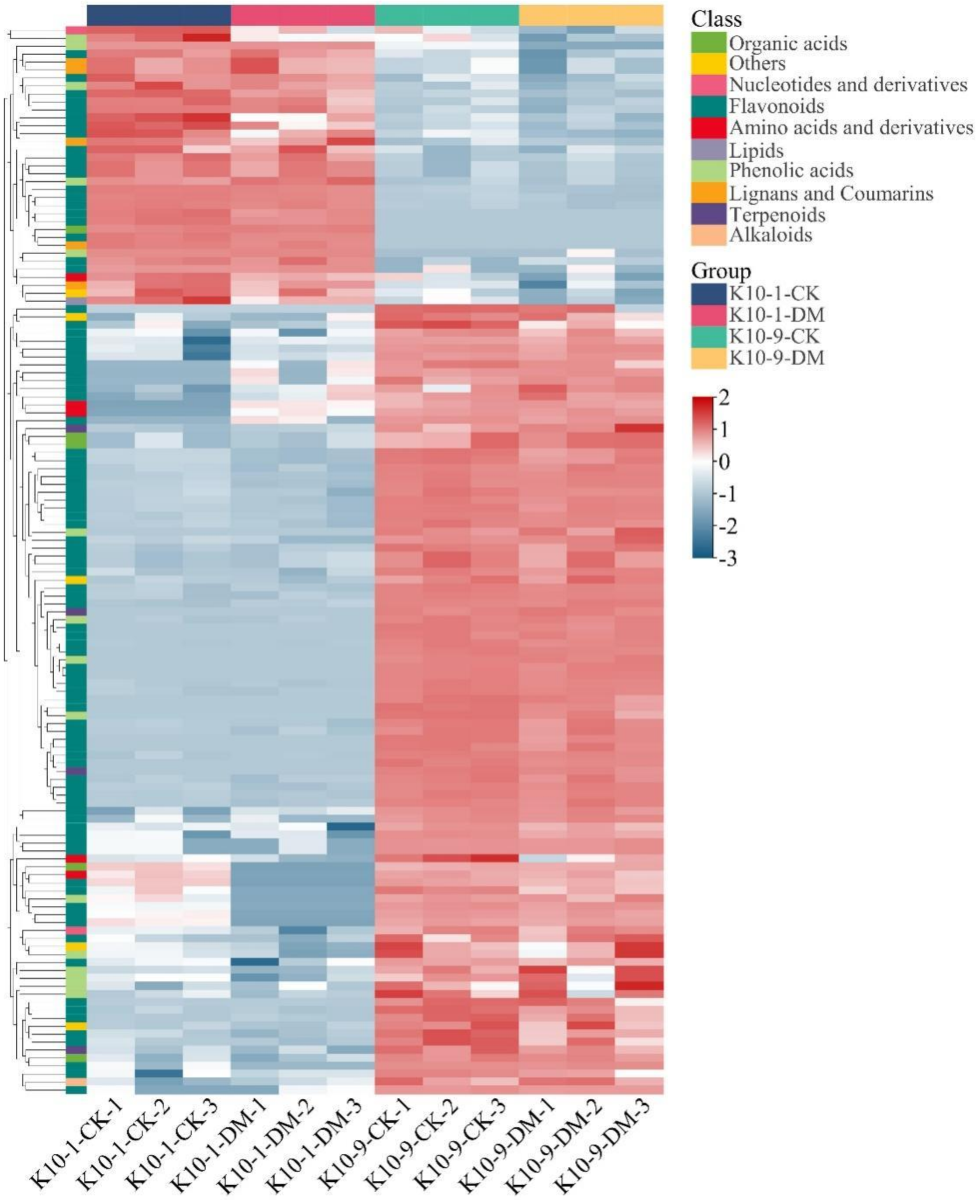

Figure S4 Heat map representation of the 35 and 99 differentially accumulated metabolites (DAMs) that consistently exhibited higher and lower contents in 'K10-1' compared to 'K10-9' in both control and treatment conditions, respectively. The accumulation patterns are based on the observations in the melon leaves of 'K10-1' and 'K10-9'.

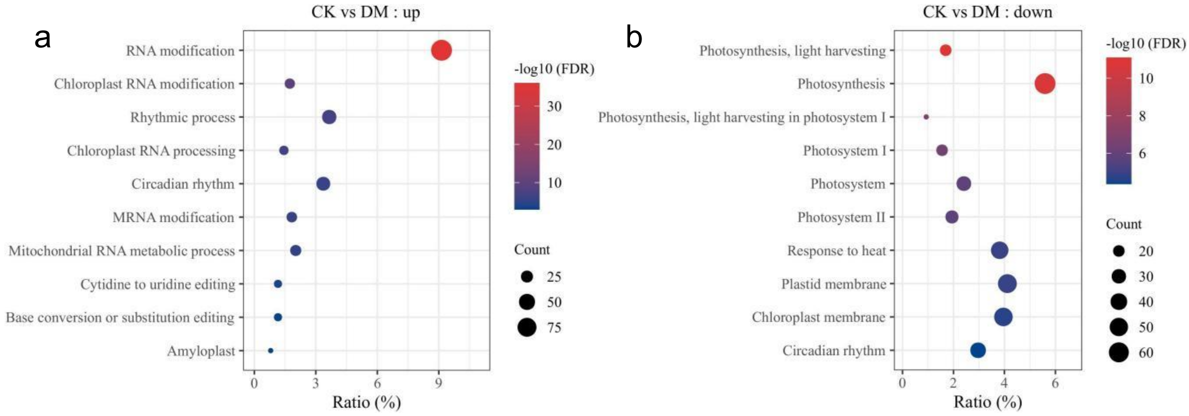

Figure S5 Top 10 GO enrichment terms for the (a) up-regulated and (b) down-regulated differentially expressed genes (DEGs) identified between the treatment and control conditions in both cultivars.

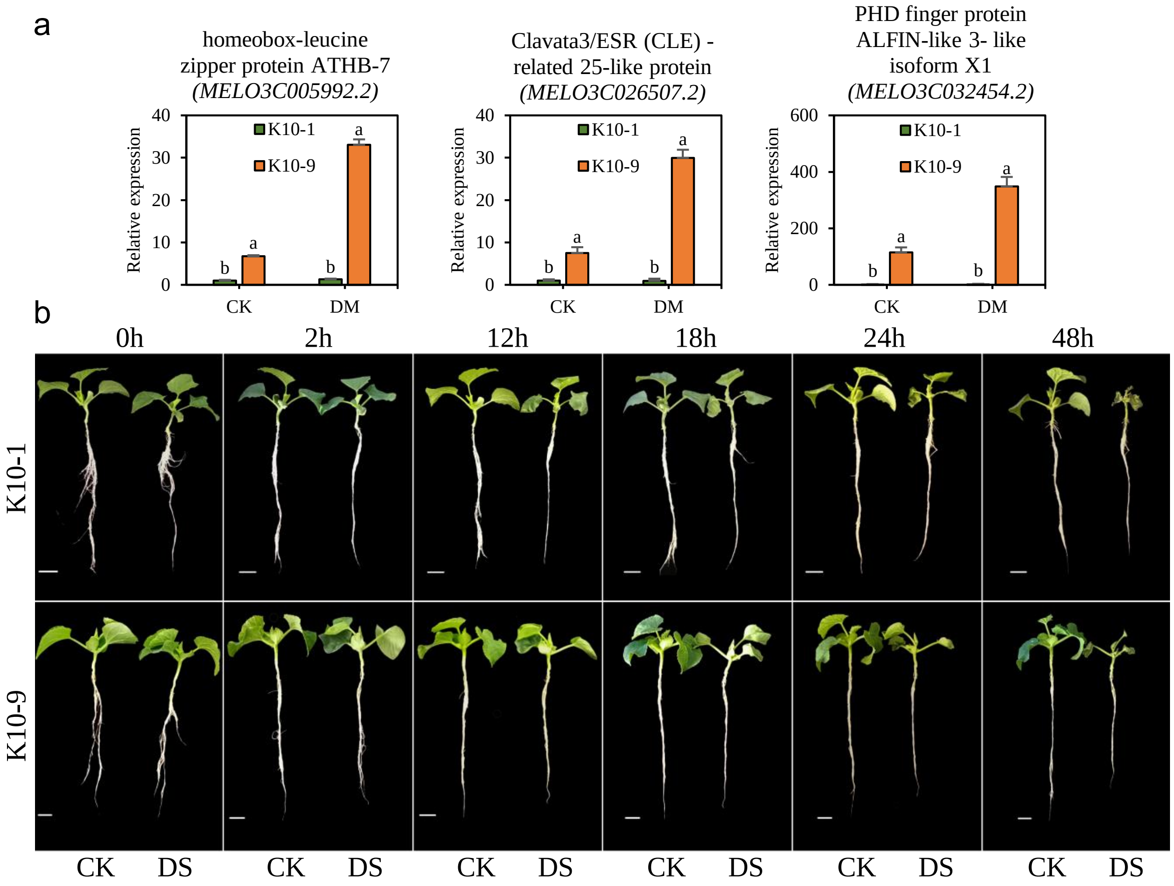

Figure S6 Effect of basic expression levels of water deprivation response-related genes on drought resistance phenotype in Melon. (a) Relative expression of genes related to water responses in 'K10-1' and 'K10-9' at 0 hours post-inoculation (CK) and 48 hours post-inoculation (DM) with *P. cubensis*. Each error bar represents the mean ± standard deviation, based on three technical and three biological replicates. Distinct letters denote significant differences ($P < 0.05$) between groups as determined by the t-test. (b) Phenotypic characteristics of melon plants exposed to simulated drought conditions using 20% polyethylene glycol-4000. CK represents the control group, while DS signifies the drought stress treatment group.

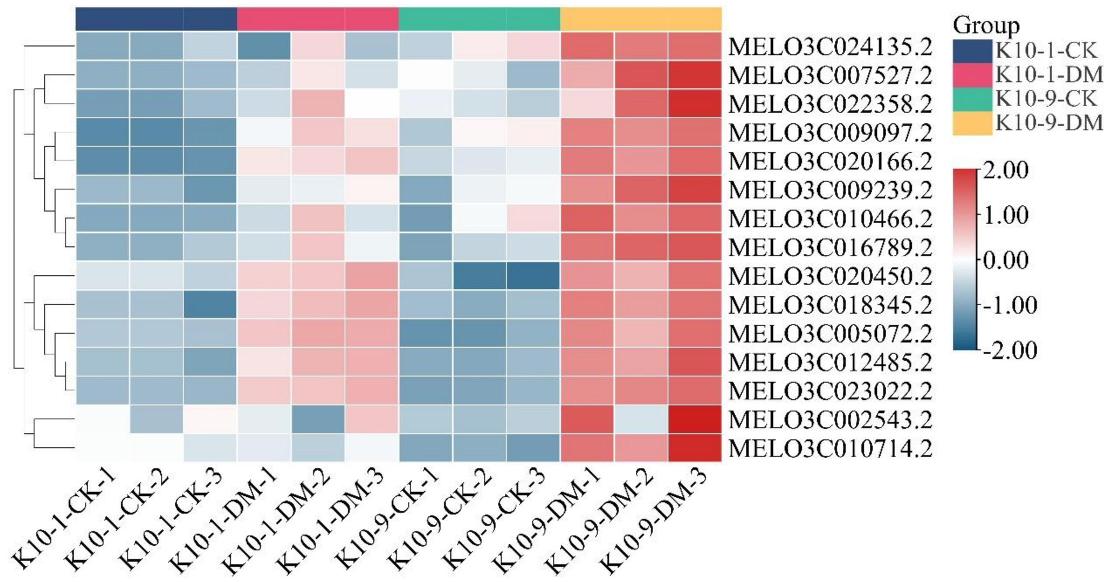

Figure S7 Heat map depicting the expression patterns of 15 salicylic acid response-related genes that are up-regulated exclusively in ‘K10-9’ following *P. cubensis* inoculation, as observed in the leaves of both ‘K10-1’ and 'K10-9' melon cultivars.

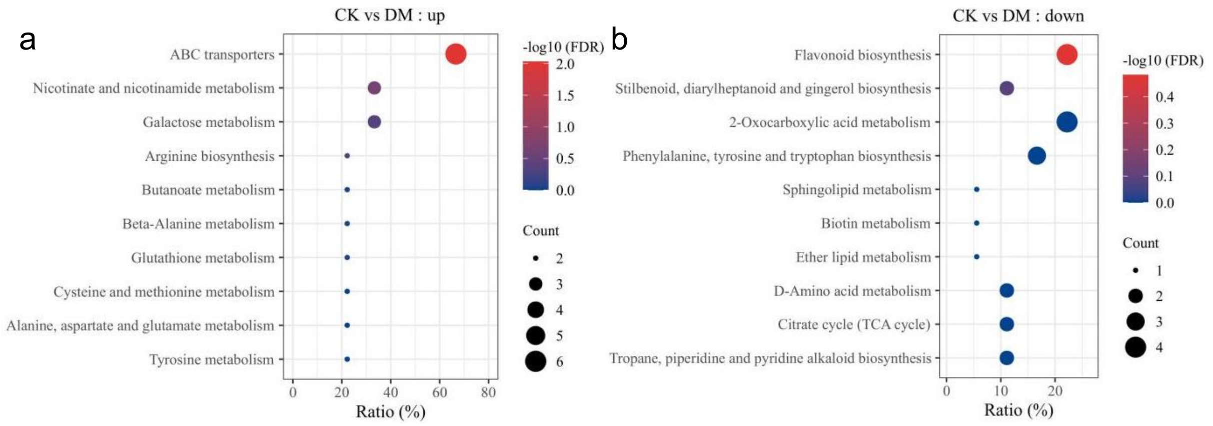

Figure S8 Top 10 KEGG enrichment pathways for a) up-regulated and b) down-regulated differentially accumulated metabolites (DAMs) identified between the treatment and control across both cultivars.

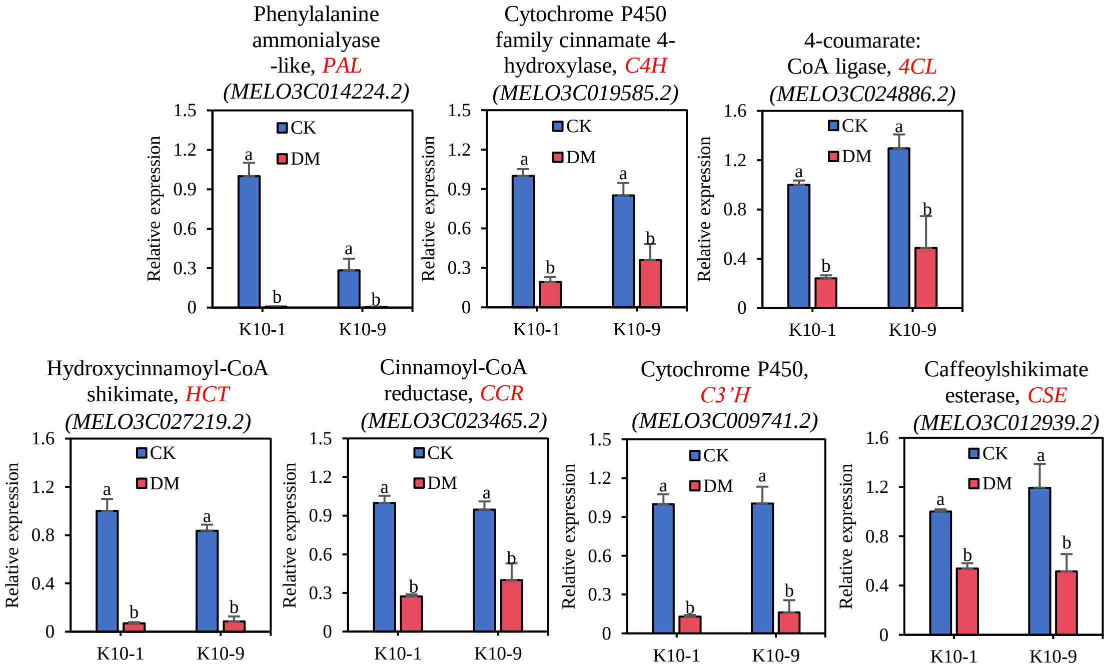

Figure S9 Relative expression levels of genes related to lignin biosynthesis in 'K10-1' and 'K10-9' at 0 (CK) and 48 hours post-inoculation (hpi) with *P. cubensis*. Error bars represent the mean ± standard deviation based on three technical replicates and three biological replicates. Groups with distinct letters signify statistically significant differences ($P < 0.05$) as determined by the t-test.
